# Expressions of emotions in minimal face perception stimuli

**DOI:** 10.1177/20416695241291648

**Published:** 2024-12-25

**Authors:** Jurģis Šķilters, Līga Zariņa, Ilze Ceple, Alina Monstvilaite, Solvita Umbraško, Santa Bartušēvica, Baingio Pinna

**Affiliations:** Laboratory for Perceptual and Cognitive Systems,; Faculty of Science and Technology, 61769 University of Latvia, Riga, Latvia; Laboratory for Perceptual and Cognitive Systems,; Faculty of Education Sciences and Psychology, 61769 University of Latvia, Riga, Latvia; Laboratory for Perceptual and Cognitive Systems,; Faculty of Science and Technology, 61769 University of Latvia, Riga, Latvia; 9312 University of Sassari, Italy

**Keywords:** face perception, eye movements, emotions, rating task, instructions, mouth, eyes

## Abstract

Face perception is considered to be a canonical example of configurational visual processing. However, not all facial information is equally important when reading facial expressions. The eyes and mouth seem to be crucial, but they seem to have different roles and significance. By varying the shape of the mouth, eyes, and other factors, we conducted two experiments: first, we examined eye movements depending on different facial configurations and different types of instructions (neutral and emotionally valenced); second, we used the same types of stimuli in a rating task. Our results indicate that the eyes provide a primary impact (when eye fixations are measured), which can be explained by the evolutionary need to establish gaze contact, but once facial expressions are observed, the mouth seems to be more significant.

Face perception is a well-known example of holistic visual processing. However, when perceiving faces, not all facial geometry seems to be equally significant. Horizontally oriented and aligned components seem to be more crucial for perceiving the face and its identity (Pachai et al., 2018). But what happens if the core components, including the mouth and eyes, are varied? Furthermore, what if the observer is looking for a particular valence in a face (compared to neutral observation)? And what is the structure of the dynamics in real-time face perception? Not all facial components are processed simultaneously, therefore, raising the question on what are the parts of the face that we fixate on more frequently, primarily, and longer by default? Finally, more generally, what are the bottom-up and top-down constraints in perceiving faces? These are the core questions that we will examine in this study.

Humans are strongly attuned to face perception (compared to other types of object perception) but also frequently perceive illusory faces containing the same or similar facial expressions or emotions as real faces. This phenomenon is called *pareidolia*, referring to the frequent ability to see faces in unrelated stimuli (Alais et al., 2021; Wardle et al., 2020).

According to recent evidence from neuroscience, face perception is a stage-wise phenomenon where we initially process illusory faces in a similar way—quickly and somewhat automatically—to real faces (Keys et al., 2021; Wardle et al., 2020, for an eye tracking study of pareidolia: Windhager et al., 2010). Even when subjects know that they are perceiving an object and not a face, they still recognize emotions, which is interesting because faces and objects are represented neurally in different ways (Wardle et al., 2020). Further, it seems that both human and illusory faces (i.e., the cases of pareidolia) share the same facial expression processing mechanism, which is evidently not strongly linked to human facial components (Alais et al., 2021). According to Alais et al. (2021), the recognition of a particular expression is not necessarily linked to facial features such as skin color or facial contours. Consequently, finely grained muscular structure is less important when recognizing some basic expressions.

A nontrivial question regarding face perception concerns the primacy of *perceptual* (physical, configurational facial features) versus *affective* (affects conveyed by these faces) factors. Some converging evidence shows that facial expressions are primarily dependent on perceptual rather than affective factors (Calvo & Nummenmaa, 2016). Further recent findings show that emotion recognition in human faces is more fine-grained than previously assumed: facial signals share different emotional reactions both on a *categorical* (referring to different emotional categories) and a *dimensional* (referring to valence and arousal dimensional space) basis (Liu et al., 2022; Mendolia, 2007; for a wider context and additional factors of valence and arousal, see also Adolph & Alpers, 2010; Sutton et al., 2019). Contextual variables, personal identity, demographic factors, and other perceptual impacts (e.g., voice), together with the perceiver's demographic factors, constrain the resulting interpretation.

Finally, an additional interrelated factor, though to some degree independent, might be human emotional sensitivity to different types of lines and shapes (Salgado-Montejo et al., 2017): line convexity seems to be linked to happiness, whereas concavity is linked with sadness and, in general, simple lines and shapes represent emotions if linked with some simplified facial features. A downward pointing “V” (eventually, in virtue of similarity to facial expressions) seems to be threatening, detected quickly and accurately in visual search tasks, and captures attention (Larson et al., 2007, 2012; Watson et al., 2011). Tipples (2007) demonstrated that the “V” eyebrow shape had the most prominent effect on determining threat, followed by the mouth shape, eye type and mouth curvature. Kohler et al. (2004) demonstrated that the recognition of different emotions (happiness, sadness, anger, etc.) can be associated to different face elements, for example, fear expressions are related to wide eyes, raised eyebrows and stretched mouth, while happiness was related to raised cheeks, tightened lower eyelid and lip corners turned upwards.

Threat-related stimuli seem to capture attention rapidly. Even in the absence of facial stimuli, simple, context-free shapes or lines representing angularity, diagonality, and curvilinearity seem to induce the perception of threat, pointing out that similar associations could be transferred also to emotion perception based on the face shape (Aronoff, 2006; Aronoff et al., 1988, 1992; Larson et al., 2009,  2012; Watson et al., 2011). Additional evidence indicates that roundedness and angularity (even without facial features) are linked to positive and negative emotions (Bar & Neta, 2006; see also Aronoff, 2006). Simple geometric shapes, V-shaped triangles, and curvilinearity might induce emotional valence and can be considered low-level threat-inducing factors (LoBue, 2014; Watson et al., 2012). Additionally, symmetry and curvature are rated positively in the case of abstract visual contours (Chuquichambi et al., 2021). Despite these studies showing that geometric factors alone might induce emotional effects, conflicting evidence (Tipples et al., 2002) shows that the perception of threat due to V-shaped components is easily detected only in conditions where other facial features are also present (i.e., rapid thread detection does not operate in low-level perception where other facial configurational effects are not provided).

To sum up, there seems to be a consensus that facial recognition is (a) stage-wise and (b) a holistic process. The detection of facial feature-like stimuli and recognizing and resolving face-like objects and faces are two stages. Although these stages might range from purely perceptual processes to the processes of recognition, resonating with existing knowledge, facial expression *and* identity information are temporally and functionally different (Adolphs, 2002). Furthermore, there is evidence that face processing unfolds in real-time situations stage-wise, indicating that gender and age emerge before the rest of one's identity (Dobs et al., 2019) and emotional information (Colombatto et al., 2021).

Finally, as argued at the beginning of this paper, face perception is *holistic*, but in several meanings: perceiving a face cannot be reduced to its constituent parts (Adolphs, 2002). In that respect, it differs from the recognition of other types of objects; parts of faces are recognized substantially less efficiently because faces seem to be recognized and processed primarily as “undifferentiated wholes”; therefore, “face recognition involved disproportionally more holistic representations than recognition of other types of patterns” (Farah et al., 1998, p. 484). The holistic representation of faces, however, does not mean that (a) the stage-wise processing and perception of faces is in all stages holistic (or equally holistic), and (b) all face regions are equally important in stimulus sensitivity. (On the problems with weak correlations between different measurements of different holistic face features, see White & Burton, 2022). Finally, holistic face perception also means an interdependency between featural and configural information (Tanaka & Sengco, 1997).

Since the human emotional sensitivity is linked to the different lines, shapes and therefore, the geometry of the visual stimulus, the aim of this study is to examine how different facial components (e.g., eyes and mouth) and their geometric variations (as represented in simple schematic line drawings) impact the perception of emotions and what is the dynamics of emotion assignment (as measured by eye tracking).

## Incomplete Visual Representations of Faces

Several studies, at least partially due to the COVID-19 pandemic, have recently been conducted to determine how face masks constrain face perception. Most evidence shows that covered faces decrease (a) the recognition of basic facial expressions (Freud et al., 2020), (b) emotion detection (also impacting the perception of closeness/proximity) (Grundmann et al., 2021), (c) speech recognition and comprehension (Giovanelli et al., 2021), (d) average perceived comfort in a female population (Howard, 2021), and (e) the relative feeling of independence among males (Howard, 2021).

However, these results do not seem to be conclusive. For example, Oldmeadow and Koch (2021) report increased trustworthiness and attractiveness and reduced racial impacts; no negative effects on mask-wearers were observed in their study. Also, Dalmaso et al. (2021) show that face masks have no negative impact on gaze cueing, which takes place independently of mask use. Other studies indicate even more complex results. For instance, Parada-Fernández et al. (2022) show that although wearing a mask indeed impairs facial emotion recognition (with the exception of surprise and happiness), it increases the average perception of attractiveness. Although masks decrease accuracy in face recognition and emotion reading, there seems to be a comparable difficulty in face perception with sunglasses (Noyes et al., 2021).

A more complex pattern of impairment (where not all emotions are impacted to the same degree) in a developmental context is provided by Carbon and Serrano (2021). Although emotion recognition was impaired in both children and adults, children displayed differences in performance for particular emotions and better emotion-reading performance in cases of angry and neutral faces when face masks were used. Furthermore, different personality features (e.g., extroversion and agreeableness) of the observer also impact emotion recognition (McCrackin et al., 2022).

Additionally, face masks have an impact on age perception by supporting age overestimation in young women; although this impact is similar to the impact of makeup, they do not seem to be additive (Davis & Attard-Johnson, 2022). Interestingly, transparent masks also seem to impact face perception—the reidentification of a person seems to be impaired, although no emotions are impacted, which could indicate that emotion perception and the recognition of facial identity are two dissociated mechanisms (Marini et al., 2021).

In general, incomplete or covered faces seem to disturb the holistic processing of facial information. According to Maurer et al. (2002) and Freud et al. (2020) (for consistent models, see also Farah et al., 1998 and Rhodes, 1988), at least three holistic processes operate on faces: (a) detecting first-order face-defining relations (shared facial features such as eyes in relation to the mouth and nose, resulting in the recognition of a face or face-like configuration), (b) binding the relations into a coherent gestalt (recognizing the particular identity of a face), and (c) resolving or establishing second-order relations (e.g., spacing and distances) between the features. When considering these three processes, it should be kept in mind that they might be interrelated and overlap. Nevertheless, the priority of overall configurational information with respect to local featural information is consistent with all approaches (for overviews, see Farah et al., 1998; Maurer et al., 2002).

However, covering the mouth seems to result in lacking a critical component for these holistic processes (Freud et al., 2020). At the same time, the relative frequency of observed covered faces also impacts the way they are perceived: frequency of occurrence seems to increase the acceptability of face coverings (e.g., masks) (Carbon, 2021). Furthermore, incomplete facial stimuli raise a question regarding the importance of separate components of facial information. V-shaped eyebrows seem to be the most important threat-inducing stimuli and are considered less pleasant than rounded or flat eyebrows (Tipples, 2007; Tipples et al., 2002). According to Tipples (2007), facial features form a particular hierarchy in cases of threat perception: eyebrow type > mouth type > eye type > mouth curvature.

## Individual Differences and Demographic Variables

Face perception is subject to substantial individual differences (White & Burton, 2022). Although recognition is only one aspect of face perception, according to recent evidence, recognition abilities seem to be linked—participants who are good at face recognition also seem to be good at voice recognition—but, at the same time, no links based on performance in face perception to general intelligence can be observed (Connolly et al., 2019; White & Burton, 2022; for a moderate effect of general intelligence see, e.g., Gignac et al., 2016).

Face perception seems to be sensitive to the demographic variables of both the observer and the represented face. Race, age, and gender are recognized surprisingly efficiently and are more foundational than the expression of emotions, which is a more culture-dependent factor (Colombatto et al., 2021). According to Colombatto et al. (2021), race and gender are recognized at an exposure of 33.33 ms, which is similar to the speed of simple face detection. This means that face detection most likely automatically and rapidly integrates demographic variables; however, worth keeping in mind that initial reaction to exposure within a particular time and the processing time can be different and the overall processing time of demographic variables might exceed the detection time and be also slower. At the same time, if sex differences among the observers are compared, female participants seem to be more positive and assign more arousing expressions to faces than male observers (Proverbio, 2017).

## Our Study

In our study, we examine minimal facial stimuli in the form of simple line drawings (Appendix A). Our aim is to explore the impact of different facial components, their geometric features and relations on the perception of facial expressions and emotions. In particular, we are testing the assumption that the mouth is the crucial factor in determining emotions from faces. Even if the types of eyes and other facial components are varied, the mouth intuitively seems to determine the overall emotion expressed by the face. The impact of the distance between eyes on configurational face perception was examined by Tanaka and Sengco (1997), whose analysis indicated that the configurational effects are primary with respect to the distance between the eyes (altering the position of the eyes impaired the recognition of nose and mouth). However, the impact of the distance between the eyes and other features on emotion reading has not previously been examined in detail.

Furthermore, we explore the question of whether verbal instructions shape the perception of particular facial emotions. If a neutral face is accompanied by an instruction to rate a happy face, does this increase the perceived happiness in the facial expression? Although there is evidence that top-down processes might impact the perception of facial expressions (e.g., according to Ying (2022), attention modulates ensemble coding), there is less evidence about the details of the impact of verbal instructions. We hypothesized that instruction-driven and valence-related attention might impact the perception of facial information in the early (eye tracking results, see Experiment 1) and later (rating task, see Experiment 2) stages of processing. Although, according to previous results (Holmes et al., 2003), attentional guiding can improve the perception of facial emotions, it is less clear what happens if different elements of facial information are modulated.

## Experiment 1: Eye Tracking

### Participants

In total, 60 participants from the Academic Center on Natural Sciences, University of Latvia (18–28 years old, *M*_age_ = 21, SD = 2 years) were enrolled in the study: 49 women (78%) and 11 men (22%). Each participant's near visual acuity (with or without contact lens correction) was at least 0.8 in decimal units. None of the participants indicated any eye, neurological, or general dysfunction affecting oculomotor performance or any other factors that could affect the study results. Participation in the study was voluntary. Participants were informed about the study methods applied, the opportunity to get acquainted with the results, and the possibility of withdrawing from the experiment at any time. The participants were not informed about the aim and the scope of the study until the end of the experiment.

### Eye Movement Analysis

Eye movements were recorded with Tobii Pro Fusion video-oculograph—a non-invasive, video-based eye tracker detecting gaze direction on a computer screen based on pupil position and the corneal reflection analysis in the eye image. The Tobii Pro Fusion device parameters applied in the current study were as follows: data acquisition frequency—250 Hz; accuracy—0.04° under optimal conditions. Stimuli were presented to participants on a Samsung S24C650PL (LCD, 23.6 in, 1920 × 1080 pixels) computer screen.

### Stimuli

**Figure 1.**
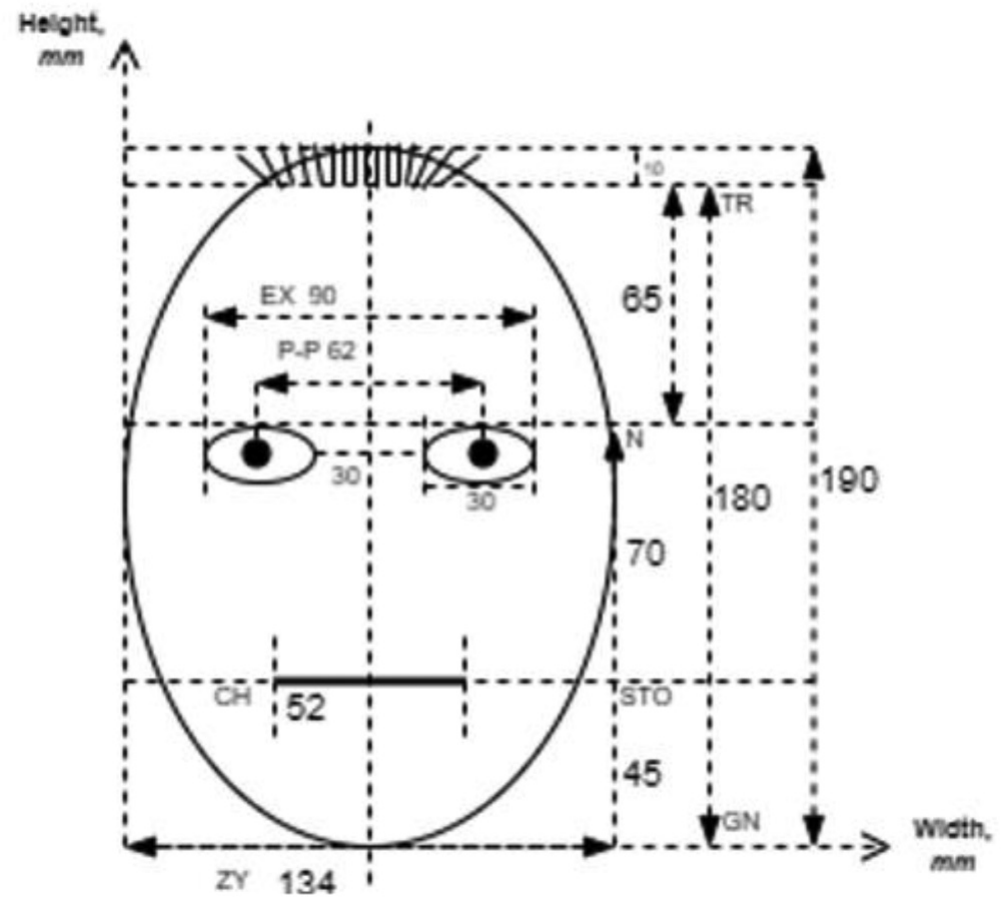
Dimensions and proportions of the reference stimulus, which were taken as a basis for creating the experimental stimuli.

Face stimuli were created based on the average anthropometric indicators of the main facial features of different European nations described by Farkas et al. (2005). These parameters were applied to create reference stimuli that were as close as possible to real facial proportions (Figure 1). A total of 54 different stimuli were created (including the reference stimuli and the stimuli where face parameters were experimentally varied) (see Appendix A). Facial features that were experimentally varied were the following: the overall shape of the face (oval according to the anthropometric measures and round [width increased by 16% and height decreased by 16% with eyes located on the diameter)], mouth size (length decreased and increased by 33% relative to the reference stimulus), mouth thickness [thin (dotted line) and thick (66% thicker than the reference)], mouth curvature (convexity and concavity decreased and increased by 50% relative to the reference), with body (according the anthropometric measures, in this case the size of face was seven times smaller than the reference), with ears (cat and rabbit ears), presence of face mask, absence of mouth, eye size [closed eyes (only lines) and wide eyes (height increased by 66% relative to the reference)], distance between the eyes (decreased and increased by 33% relative to the reference), and distance between the eyes and mouth (decreased and increased by 33% relative to the reference). The extent of the changes was designed to maintain proportions close to the natural facial structure and expressions. All stimuli represented one of three facial expressions: happy, sad, or neutral. The change in expression was based only on mouth shape. The total size of each stimulus in the experiment was 11° or 19 cm.

### Procedure

All participants (*n *= 60) were randomly arranged into three equally sized groups (*n *= 20 ea.). Participants in the first group were instructed to observe the stimuli without any specific instructions. The second group was instructed to “look at the happy faces,” and the participants attended only faces with happy or neutral emotions. The third group was instructed to “look at the sad faces,” and the participants attended only faces with sad or neutral emotions.

The participants sat at a distance of 0.65 m from the screen, which was positioned at the eye level. All participants were instructed to look at the stimuli with both eyes without any head motion. The participants were then given an additional task according to their group.

Before starting the eye movement recordings, each participant performed a nine-point calibration process determining the coordinates of their pupil's center and corneal reflex in the eye image at different viewing directions. After a successful calibration (accuracy of 0.75° or higher), the participants attended the experimental stimuli.

**Figure 2.**
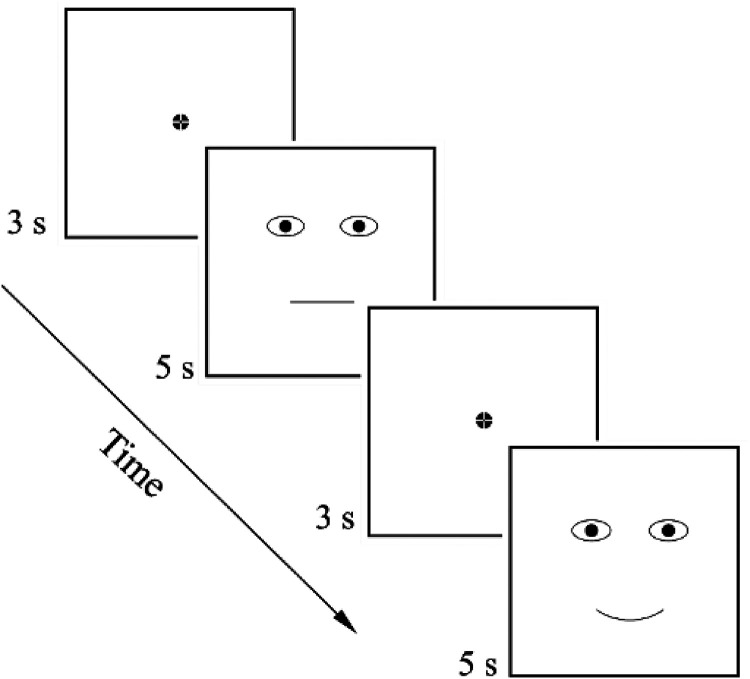
Arrangement of stimuli demonstration.

Each stimulus was presented on the computer screen (one after the other) for 5 s, and the participants had to fixate their gaze on the displayed stimuli according to the instructions given prior to the experiment. The fixation cross was displayed for 3 s at the center of the screen (in the stimulus’ nose area, equidistant from the eyes and mouth) before each stimulus presentation (see Figure 2). Stimuli were presented in a random order.

The participants in the first group attended all 54 stimuli (instead of 36 stimuli demonstrated to the second and third group of participants), and the total experiment time for the first group was around 7 min. Considering that prolonged monotonous looking at the stimuli might impact the study results, tasks unrelated to the purpose of the study (fillers) were included between the stimuli demonstrated to the first group, thus shortly distracting the participants’ attention. These tasks included reading a sentence and evaluating whether it sounded like it was said by a native speaker. A total of four fillers were inserted: two at a time after 18 stimuli had been demonstrated. There was no time limit for the participant to respond.

**Figure 3.**
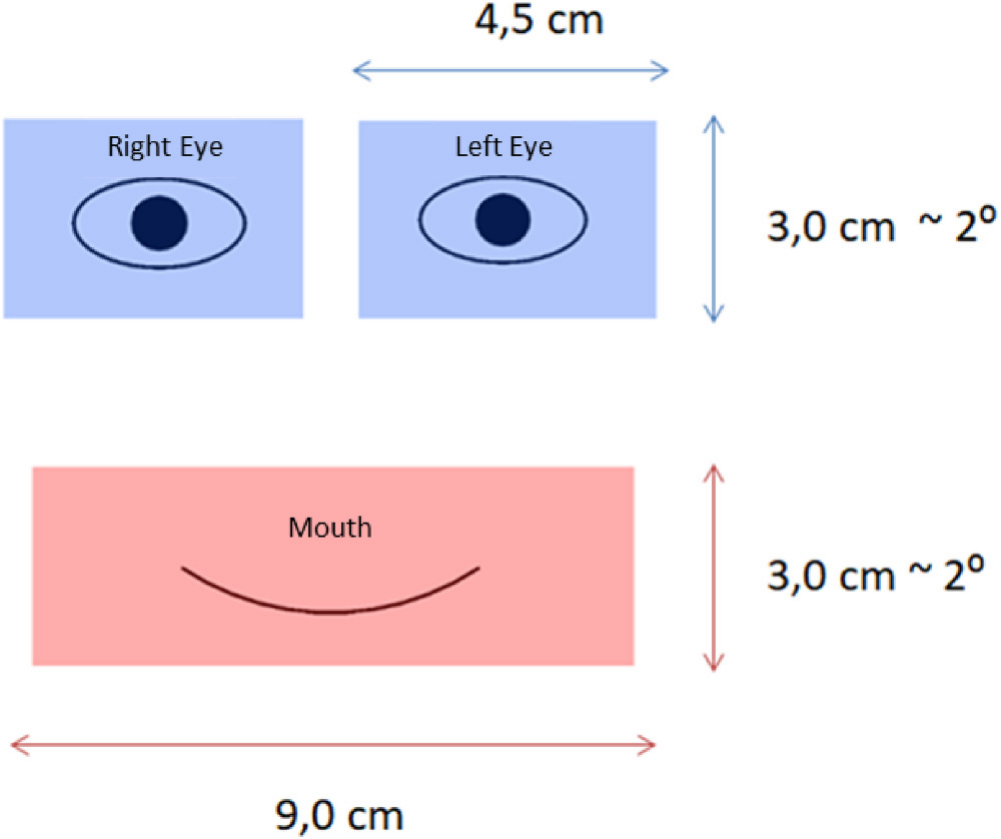
Areas of interest (AOIs) in the eye movement analysis.

Eye movement analysis was performed using data from two specific areas of interest (AOIs): the eyes and the mouth (Figure 3). The area of each eye was 4.5 × 3.0 cm, and the area of the mouth was 9.0 × 3.0 cm. Such AOI dimensions were selected to cover the entire eye and mouth area, considering the calibration accuracy (the accuracy was within 0.26–0.74° or 0.31–0.92 cm) and the fact that participants might fixate on the outer contour of the eyes or mouth instead of the center of the element. The total size of the areas of both eyes was equal to the size of the mouth area. The following parameters of eye movements were included in the subsequent data analysis: the direction of the first saccade, the total duration of fixations on the AOIs, and the total number of fixations on the AOIs.

### Results

#### Effect of the Instructions on the eye Movement Parameters

**Figure 4.**
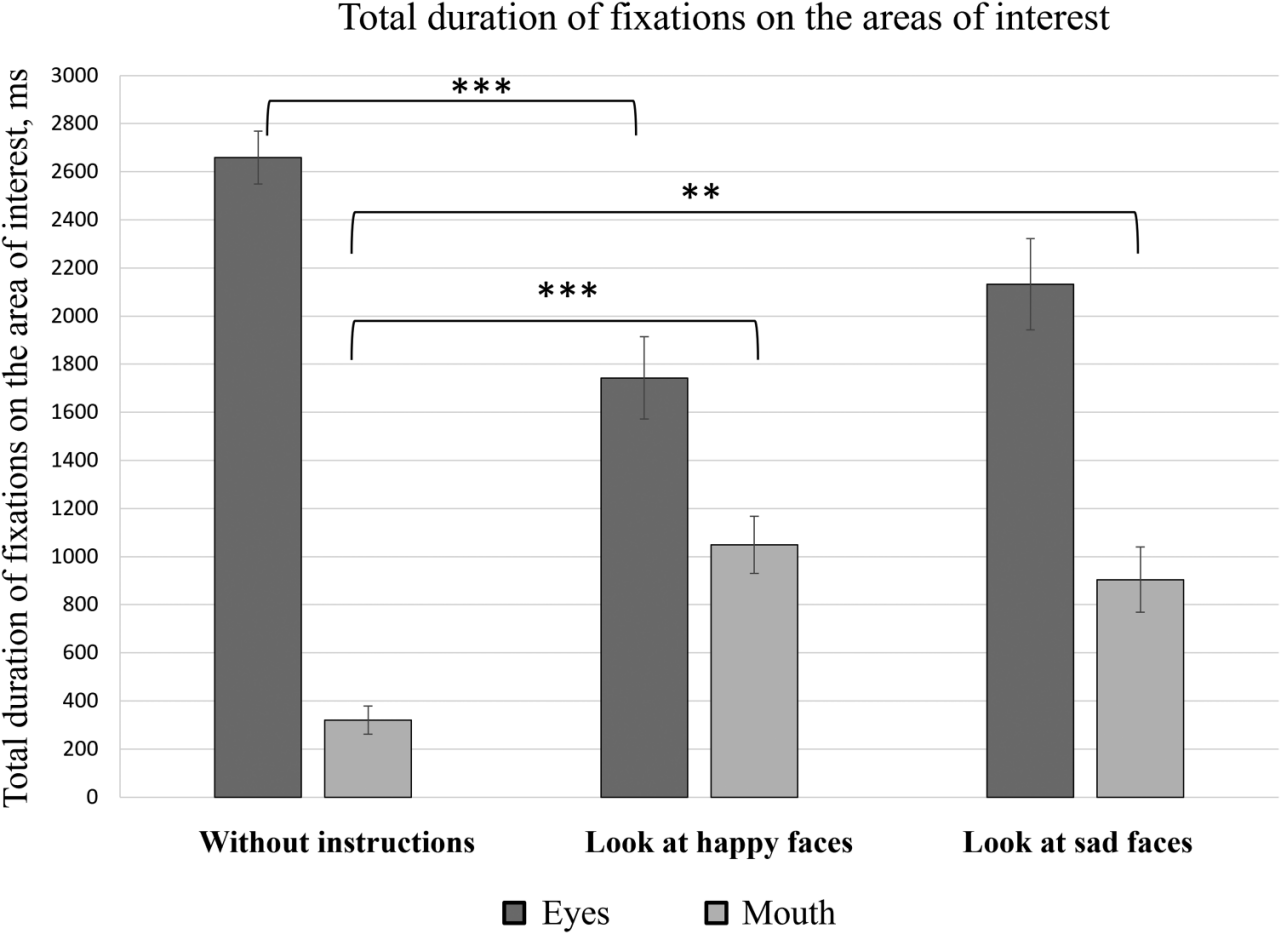
Total duration of fixations on different AOIs represented by the mean value and standard errors of all participants. *p ≤ .05, **p ≤ .01, and ***p ≤ .001.

Eye movement data were analyzed with RStudio and IBM SPSS Statistics software. The initial results of total fixation duration indicate that regardless of the given instructions (no instructions; instructions to “look at happy faces”; instructions to “look at sad faces”), participants fixated on the areas of both eyes for a longer time than the mouth area (Figure 4). Since the number of facial stimuli was different based on the instructions’ conditions, the median values of the total fixation durations on all the facial stimuli were selected for further data analysis. A one-way analysis of variance (ANOVA) indicated statistically significant differences in total fixation durations between the eye area (*M *= 2659 ± 495 ms) and mouth area (*M *= 320 ± 262 ms) in the case of no specific instructions [*F*(1, 38) = 348.90, *p *< .001]; in case of instructions to “look at happy faces” [eyes: *M *= 1743 ± 762 ms; mouth: *M *= 1049 ± 531 ms; *F*(1, 38) = 11.2, *p *= .002]; and in the case of instructions to “look at sad faces” [eyes: *M *= 2132 ± 847 ms; mouth: *M *= 904 ± 606 ms; *F*(1, 38) = 27.78, *p *< .001].

The total fixation time on the mouth area was longer when the participants were instructed to attend to faces with certain emotional expressions than when faces were attended with no specific instructions (Figure 4). To formally test this observation, a one-way ANOVA was performed, which confirmed the statistically significant effect of instruction on the total fixation time on the mouth area [*F*(2, 57) = 12.45, *p *< .001]. Post-hoc analysis (Tukey's HSD) showed significant differences between the total duration of fixations on the mouth area in the case of no specific instructions (*M *= 320 ± 262 ms) and when the participants were instructed to “look at happy faces” (*M *= 1049 ± 531 ms) (*p *< .001). A significant difference in fixation durations on the mouth area was also observed when comparing participants who attended the stimuli with no specific instructions (*M *= 320 ± 262 ms) with those who were instructed to “look at sad faces” (*M *= 904 ± 606 ms) (*p *= .001). Furthermore, when the participants were instructed to “look at sad faces,” the total fixation duration (*M *= 904 ± 606 ms) was smaller than when the participants were instructed to “look at happy faces” (*M *= 1049 ± 531 ms); however, in this case, the observed difference was not statistically significant (*p *= .621).

Similarly, the total duration of fixations on the area of both eyes was shorter when the participants were instructed to attend the stimuli expressing certain emotions than when participants looked at the stimuli without any instructions (Figure 4). A one-way ANOVA was performed as a formal test of these observations. The results of the statistical analysis demonstrated a statistically significant instruction effect on the total time spent on the area of both eyes [*F*(2, 57) = 8.22, *p *= .001]. Post-hoc analysis (Tukey's HSD) indicated statistically significant differences between the total duration of fixations on the area of both eyes in the case of no specific instructions (*M *= 2659 ± 495 ms) and when the participants were instructed to “look at happy faces” (*M *= 1743 ± 762 ms) (*p *< .001). No significant differences were observed when comparing the group of participants with no specific instructions (*M *= 2659 ± 495 ms) and the group of participants who were instructed to “look at sad faces” (*M *= 2132 ± 847 ms) (*p *= .06), nor when comparing the groups of participants who were given the instructions to “look at sad faces” (*M *= 2132 ± 847 ms) and to “look at happy faces” (*M *= 1743 ± 762 ms) (*p *= .209).

**Figure 5.**
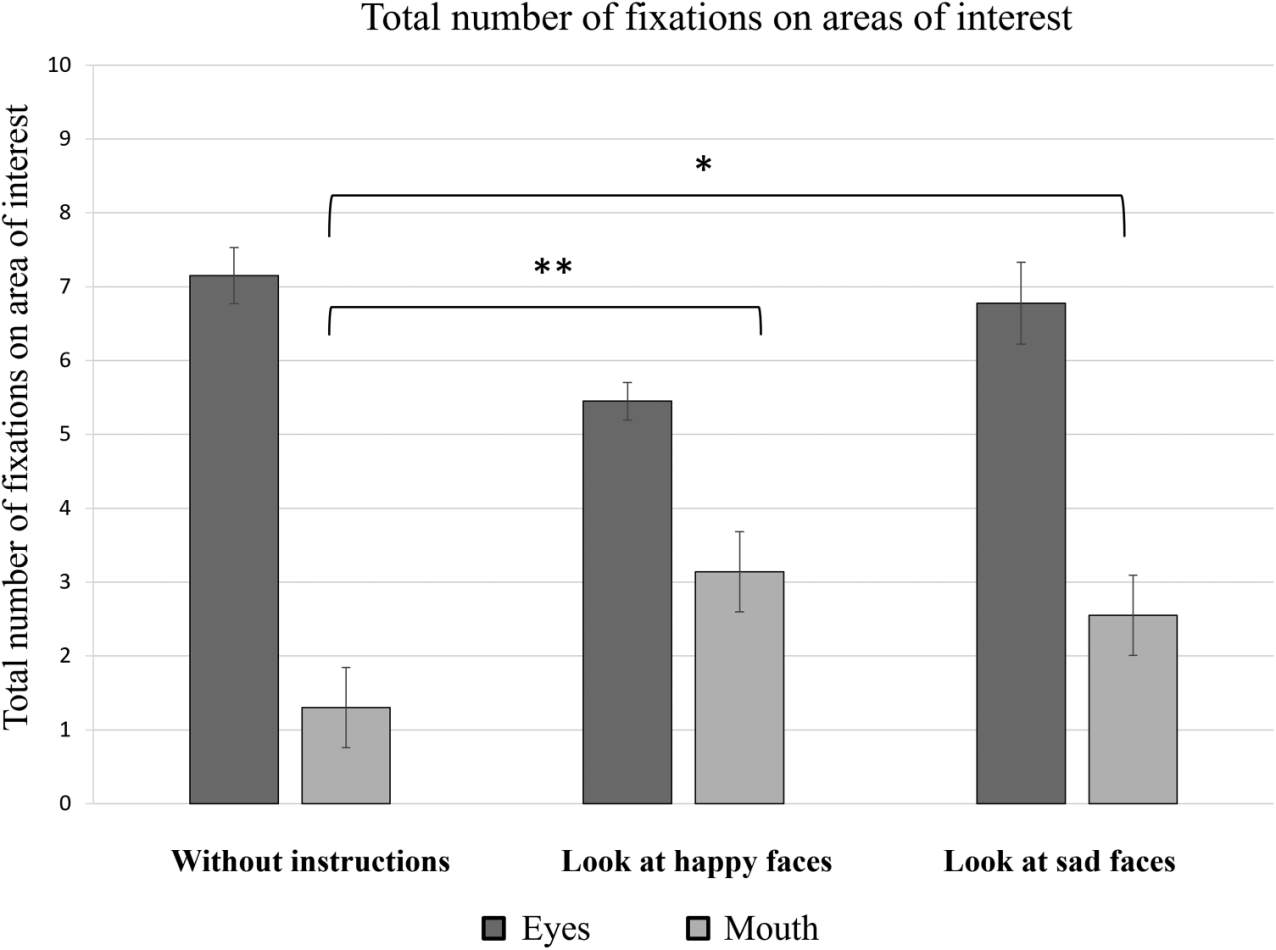
Total number of fixations on different AOIs represented by the mean value and standard errors of all participants. *p ≤ .05, **p ≤ .01, and ***p ≤ .001.

Further data analysis involved evaluating the average number of fixations on the areas of the eyes and mouth based on the different instructions given to the participants (Figure 5). The results of a one-way ANOVA demonstrated a statistically significant difference between the number of fixations on the area of the eyes (*M *= 7 ± 2) compared to the mouth area (*M *= 2 ± 1) in the case of no specific instructions [*F*(1, 38) = 183.71, *p *< .001]; in case of instructions to “look at happy faces” [eyes: *M *= 6 ± 2; mouth: *M *= 4 ± 2; *F*(1, 38) = 10.19, *p *= .003]; and in the case of instructions to “look at sad faces” [eyes: *M *= 7 ± 3; mouth: *M *= 3 ± 2; *F*(1, 38) = 21.03, *p *< .001].

It was also observed that the total number of fixations on the mouth area was higher when the participants were asked to attend the faces representing certain emotional expressions than when attending stimuli without any instructions. This statistically significant instruction effect was confirmed by a one-way ANOVA [*F*(2, 57) = 6.61, *p *= .002].

In order to determine which instructions were related to significant differences in the total number of fixations on the mouth area, Tukey's HSD test was applied. The results of this post-hoc analysis indicated significant differences between the total number of fixations on the mouth area when viewing the stimuli with no specific instructions (*M *= 1 ± 1) and when the participants were instructed to “look at happy faces” (*M *= 3 ± 2) (*p *= .003), as well as when viewing the stimuli with no specific instructions and when participants were instructed to “look at sad faces” (*M *= 3 ± 2 ms) (*p *= .020). When comparing the total number of fixations on the mouth area between participants given the instructions to “look at happy faces” and those instructed to “look at sad faces,” no statistically significant difference was observed (*p *= .767).

When evaluating the total number of fixations on the eye area according to instructions given (no instructions; “look at happy faces”; “look at sad faces”), a one-way ANOVA did not indicate a statistically significant effect (*p = *.127).

Regardless of the instructions given, it was observed that more than 75% of all saccades were directed toward the eye area (Table 1). However, the presence of specific instructions to “look at happy faces” or “look at sad faces” made the proportion of the first saccade toward the eyes area decrease from 85% to 75% and the proportion of the first saccade toward the mouth area increase from 15% to 25%.

**Table 1.** First saccade direction when looking at face stimuli, %.

Instruction	Eyes area	Mouth area
No instruction	85	15
Look at the happy faces	75	25
Look at the sad faces	76	24

#### The Effect of Facial Features on Eye Movement Parameters

In the further data analysis, the effect of different facial parameters (face shape, mouth size, the distance between the eyes, etc.) on the results of saccadic eye movements was evaluated. To compare the effects of different facial parameters and the effects of the faces’ emotional expressions (happy, sad, or neutral) on the total duration of fixations on the areas of eyes and mouth, a two-way repeated measures ANOVA was performed for each group of instructions. When comparing the total fixation duration times on the eye area, a statistically significant effect of different facial features was observed when no specific instructions were provided [*F*(15, 285) = 24.66, *p *< .001, *η^2^_p _*= .57]; however, no significant effect was observed when the three groups of different face emotions were compared (*p *> .05). A similar trend was observed for the mouth area: a two-way repeated measures ANOVA indicated a statistically significant effect of facial parameters [*F*(15, 285) = 9.45, *p *< .001, *η^2^_p _*= .33]; however, no statistically significant differences depending on the expression of facial emotions were observed (*p *> .05).

In order to evaluate the effect of each parameter when attending the face stimuli, multiple comparisons of the total duration of fixation indicators were performed between the reference stimulus and different face parameters using a two-way repeated measures ANOVA. Considering the relatively small sizes of the participant groups and the large number of stimuli variables (1 reference and 19 different facial parameters), the effect power of each facial variable parameter was not interpreted based on a certain critical *p*-value but ranked in ascending order by the effect size. Therefore, it was possible to assess overall trends in facial factors that have a higher or lower effect on the total duration of fixations on the mouth and eye areas.

All variable parameters of the stimuli were grouped into three categories according to their location (“the variable parameters of the eyes,” “the variable parameters of the mouth,” and “other variable parameters”). The variable parameters of the eyes were eye width and the distance between the eyes. The variable parameters of the mouth were mouth thickness, mouth curvature, mouth size, eye-mouth distance, face without a mouth, and face with a face mask. Other variable parameters were the shape of the head, face with a body, and face with animal features.

**Figure 6.**
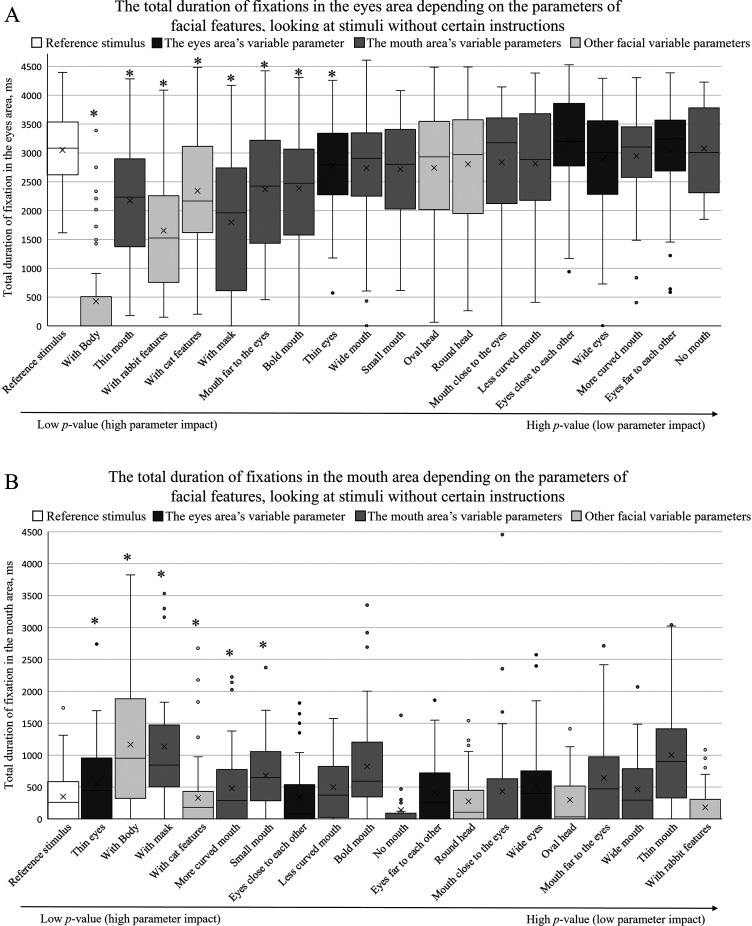
The total duration of fixations depending on facial feature parameters (A on the eye area; B on the mouth area) when no specific instructions were given. The box plots are arranged by the size of the p value in an ascending order (pairwise comparisons between the reference stimulus and the specific feature, *p < .05).

**Figure 7.**
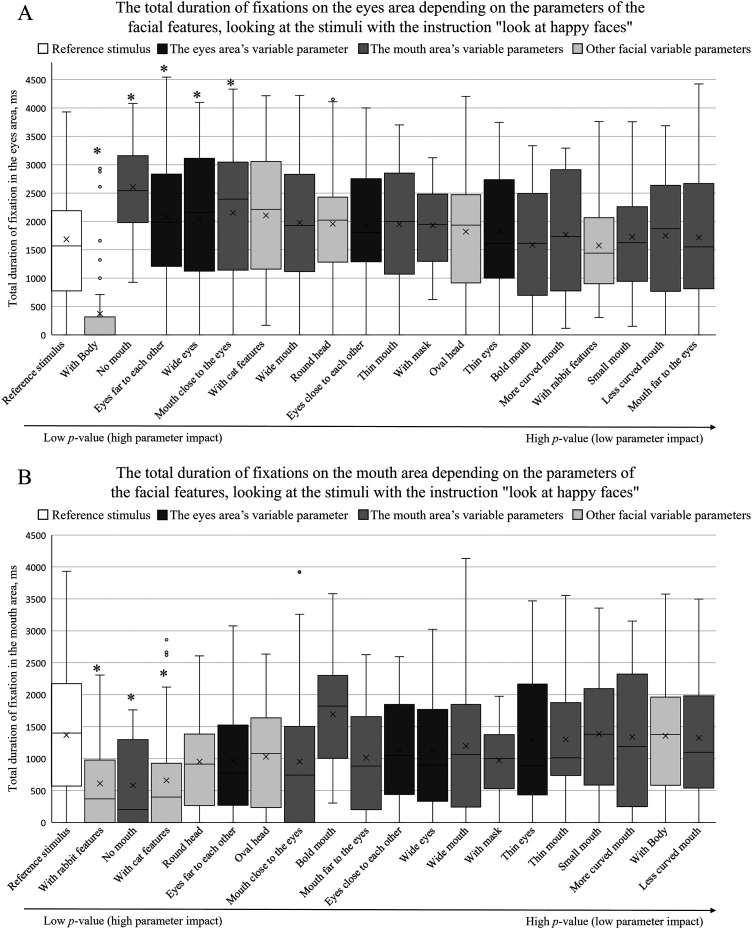
The total duration of fixations depending on facial feature parameters (A on the eye area; B on the mouth area) when participants were instructed to “look at happy faces.” The box plots are arranged by the size of the *p* value in an ascending order (pairwise comparisons between the reference stimulus and the specific feature, *p < .05).

**Figure 8.**
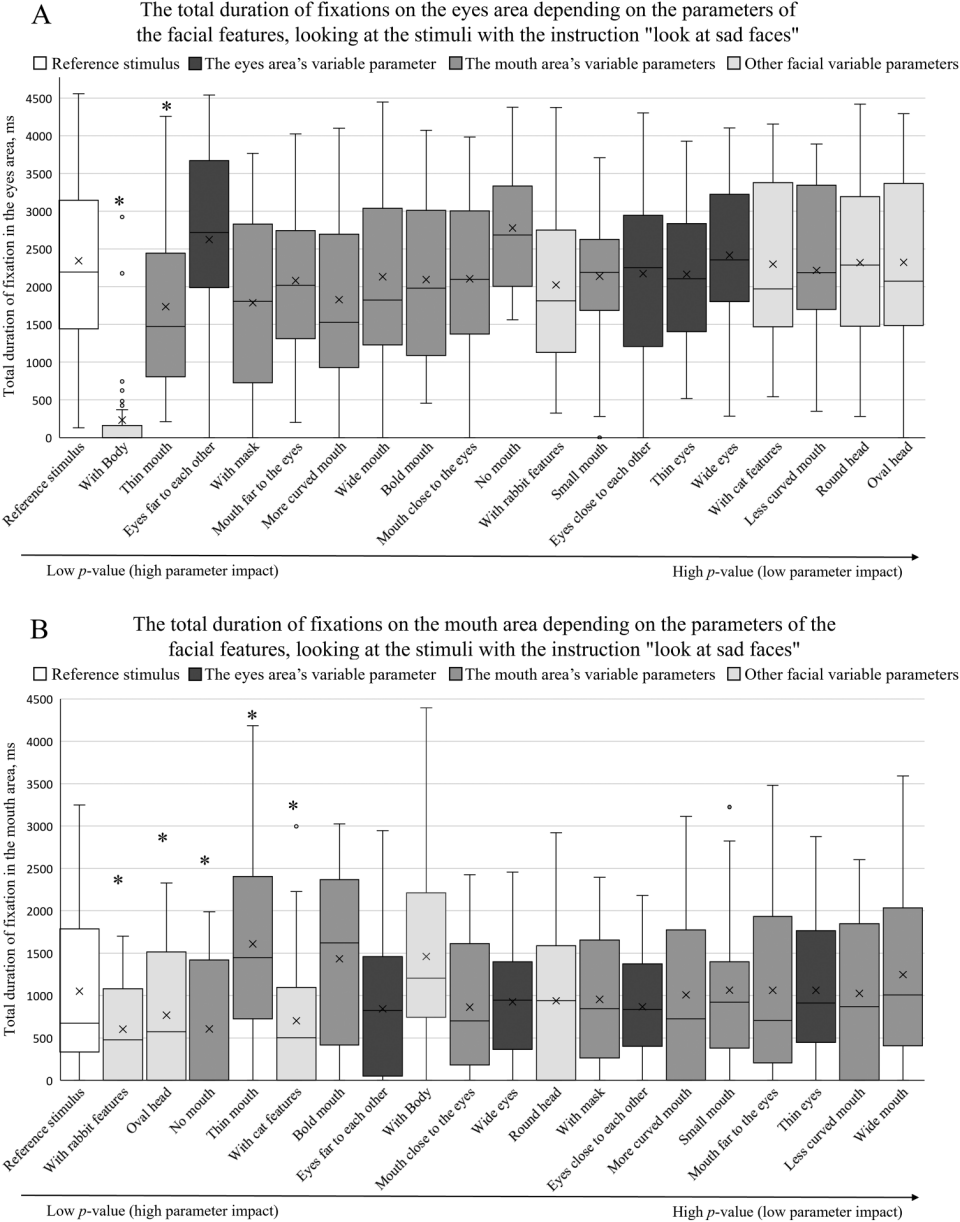
The total duration of fixations depending on facial feature parameters (A on the eye area; B on the mouth area) when participants were instructed to “look at sad faces”. The box plots are arranged by the size of the p value in an ascending order (pairwise comparisons between the reference stimulus and the specific feature, *p < .05).

Figures 6–8 summarize the total fixation duration measurements on the eye and mouth areas when viewing the reference stimulus and other stimuli with different facial parameters. The obtained results were arranged in ascending order based on the *p*-value of pairwise comparisons between the reference stimulus and each parameter. The *p*-value increases toward the right side, indicating that the effect of a certain parameter decreases. An asterisk (*) above the box plot indicates a significant difference in total fixation duration between the reference stimulus and the facial parameter.

Figure 6A demonstrates the effect of different facial parameters on the total fixational duration on the area of the eyes when no specific instructions were provided. The results do not mark any specific tendencies or differences between the three groups of variables—the results are scattered and do not indicate that by modifying only the eye, mouth, or other parameters, we could observe a higher effect on the total fixation duration when observing the eye area.

**Figure 9.**
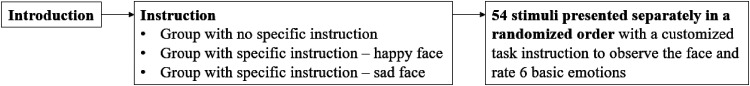
Procedure of Experiment 2.

Similarly, no clear trends were observed when inspecting the effect of all three variable groups on the total fixation duration on the mouth area (Figure 6B). Interestingly, when comparing the individual differences between the reference stimulus and other facial stimuli, there was a clear effect from the parameter “with body”: the total duration of fixations on the eye area significantly decreased, and the total duration of fixations on the mouth area significantly increased. A similar trend was observed when a face “with a mask” was shown: the total duration of fixations on the eye area significantly decreased, and the total duration of fixations on the mouth area significantly increased.

When the participants were given the instructions to “look at a happy face,” a two-way repeated measures ANOVA indicated a statistically significant effect of face parameters on the total duration of fixations when viewing the eye area [*F*(15, 285) = 7.81, *p *< .001, *η*^2^*_p _* = .29]. Similarly to the group given no instructions, the total duration of fixations was not significantly affected by the different expressions of emotion (*p *> 0.05). A similar trend was observed when viewing the mouth area: a two-way repeated measures ANOVA indicated a statistically significant effect of different facial parameters [*F*(15, 285) = 4.25, *p *< .001, *η^2^_p _*= .18]; however, no significant effect of facial expressions was observed (*p *> .05).

Figure 7A demonstrates the effect of different facial parameters on the total duration of fixations on the eye area when comparing the reference stimulus and all other facial factors. When the participants were given the instructions to “look at happy faces,” no specific trends of the different variable groups were observed with respect to total fixation duration measurements. As was the case with participants with no specific instructions, the total fixation duration on the eye area significantly decreased when a “whole body” condition was added.

Figure 7B demonstrates the effect of different facial parameters on the total duration of fixations on the mouth area, when the participants were instructed to “look at happy faces.” Here, an increased effect of other variable parameters was observed (lower *p*-values compared to other groups of parameters). A decreased effect of the eye area's parameters on the total duration of fixations on the mouth area was also observed (higher *p*-values). When comparing the individual differences of the total fixation duration measurements on the areas of eyes and mouth between the reference stimulus and different facial parameters, in the case of the condition without a mouth (“no mouth” parameter), the total duration of fixations increased in the eye area and decreased in the mouth area.

When the participants were instructed to “look at sad faces,” a two-way repeated measures ANOVA indicated a statistically significant effect of different facial parameters on the total duration of fixations when viewing the eye area [*F*(15, 285) = 14.55, *p *< .001, *η^2^_p _*= .43]. Similarly to other participant groups, there was no significant effect from differing facial expressions (*p *> .05). A similar trend was observed when analyzing the total fixational duration on the mouth area depending on the facial parameters and facial expressions: a two-way repeated measures ANOVA indicated a statistically significant effect of facial parameters [*F*(15, 285) = 5.48, *p *< .001, *η^2^_p _*= .22] and no significant effect of facial expressions (*p *> .05).

Figure 8A demonstrates the effect of different facial parameters on the total duration of fixations on the eye area when the participants were instructed to “look at sad faces.” Here, the “variable parameters of the mouth” were observed to have a higher effect in directing the gaze when comparing the results of the reference stimulus and the three groups of variable parameters. The effect of “variable parameters of the eyes” and “other parameters” on the total fixation durations on the eye area was lower and did not demonstrate any specific trends. The parameter “with body” showed the largest effect (the largest decrease in the total duration of fixations on the eye area), which was also observed in the cases of other instructions.

Figure 8B demonstrates the effect of different facial parameters on the total duration of fixations on the mouth area when the participants were instructed to “look at sad faces.” Here, no specific trends were observed: the groups of different parameters (“variable parameters of the eyes,” “variable parameters of the mouth,” “other variable parameters”) are not concentrated on one or other side of the graph.

When analyzing the individual differences in the total fixation durations between the reference stimulus and facial parameters, the “thin mouth” parameter demonstrates a significantly lower total fixational time on the eye area and a significantly higher effect on the mouth area.

As mentioned previously, the variable parameters marked with an asterisk in Figures 6–8 indicate a significant difference (*p *< .05) in the total fixational times between the reference stimulus and a specific variable parameter. These thus provide an opportunity to observe the overall effect of different facial parameters between different instruction types. We propose that in the case of no specific instructions, the overall effect of the different variable parameters was larger than when the stimuli were attended as happy or sad faces, that is, more parameters indicate a significantly smaller *p*-value compared to the reference stimulus.

To sum up, we can draw two main observations from the current eye-tracking experiment. First, regardless of the instructions, eyes play a general role in face perception: in more than 75% of all observations, the first saccade is directed toward the eye area, and the average fixation duration on the eyes is longer compared to the mouth area. Second, when the participants are given specific instructions to observe a happy or a sad face, we find increased interest in the mouth area: a higher proportion of the first saccades are directed toward the mouth, and the average fixation duration and the number of fixations on the mouth area tend to increase. This indicates the importance of the mouth's expression when analyzing facial emotions.

When the facial features (eyes, mouth, and other features) are altered, we can observe that these alterations are more significant in the case of no instructions: a higher number of parameters significantly change the total fixation duration on the eyes and mouth compared to when the participants are given specific instructions to observe happy or sad faces. Two clear trends are evident regarding the three different groups of facial features. First, when the participants are given instructions to observe a happy face, there is a decreased interest in the mouth area when different parameters of the group “other” (i.e., head shape, distance between eyes and mouth, etc.) are altered. Second, when the participants are given instructions to observe a sad face and the mouth parameters are altered, there is a decreased interest in the eye area, once again indicating the crucial role of mouth parameters when analyzing facial emotions.

## Experiment 2: Rating Task

### Participants

In total, 132 participants took part in the study (48 male, 79 female, 5 unspecified, age *M *= 30.3, *SD *= 13.7). Some were students from the Faculty of Computing, University of Latvia, while others were selected from a stratified participants’ database of the Laboratory of Perceptual and Cognitive Systems at the Faculty of Computing, University of Latvia. All participants took part in the study voluntarily, and they were informed about the data arrangement policy, the opportunity to get acquainted with the results, and the possibility of withdrawing from the experiment at any time.

### Stimuli and Study Design

We conducted an online quasi-experiment in the form of a questionnaire combined with an experimental rating task using the QuestionPro platform. It had to be completed on a computer or tablet.

After a short introduction to the study procedure, participants were asked to evaluate their emotional state according to the Swedish Core Affect Scale (Västfjäll et al., 2002). Next, participants received instructions on the experimental task and were presented with 54 face stimuli in a randomized order. Their task was to rate six classical emotions (see also Liu et al., 2022)—happiness, sadness, disgust, surprise, anger, and fear—in a randomized order. We used the same stimuli that were used in experiment 1: faces with real facial proportions that are varied with respect to facial expression (neutral, positive, negative, and without mouth) and features (mouth, eyes, and other) (see Appendix A, Table 2 shows the reference stimuli).

**Table 2.**
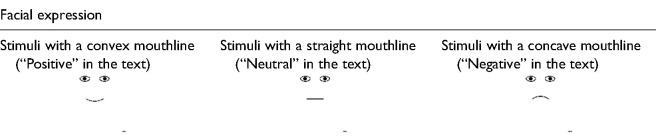
Reference stimuli.

To test the effect of instructions (and, therefore, the effect of expecting particular emotions), participants were randomly assigned to three experimental groups with different instructions (gender proportions and ages were similar in all experimental groups; although the number of participants was higher in the group with no specific instruction, the unbalanced size of the groups did not affect the overall results of analysis and the number of participants was sufficient in each group to run a comparative analysis). Two groups had specific instructions before the block of stimuli evaluation questions: “Rate whether and to what extent the faces that will be shown next are happy!” (*n *= 27) and “Rate whether and to what extent the faces that will be shown next are sad!” (*n *= 27). The third group had neutral instructions: “Next, you will see images which you have to observe and evaluate carefully” (*n *= 78) (Figure 9). The instruction was presented on a blank screen before the rating task started. In the case of specific instructions, the tasks were to rate (1) whether the presented face is happy (or sad) and (2) whether the other characteristics mentioned below [disgust, surprise, anger, fear, and sadness (happiness)] apply to it and, if so, to what extent they are expressed in the picture to be rated. The intensity scale for each of the six emotions was as follows: 0—no, 1—very weak, 2—weak, 3—average, 4—strong, and 5—very strong.

In the case of neutral instructions, the task was to rate whether the following features are visible in the image and to what extent they are expressed. Finally, participants had to answer a few demographic questions about their age, gender, and field of education/occupation. The approximate time needed to fill in the questionnaire was 30 min (mode = 29.3, mean = 33.0).

### Data Analysis

Data analysis was carried out in R and IBM SPSS Statistics 22 software. We used non-parametric methods because of the ordinal scale measurements in the experiment. The results of participants’ emotions regarding the Swedish Core Affect Scale were compared with the Kruskal–Wallis test. The Friedman test and Wilcoxon pairwise comparison tests with Bonferroni correction were used to test the impact of the features of facial geometry by experimental group and emotional state. Differences between experimental groups and genders were tested using the Kruskal–Wallis and Mann–Whitney tests and GLM univariate analysis.

### Results

**Figure 10.**
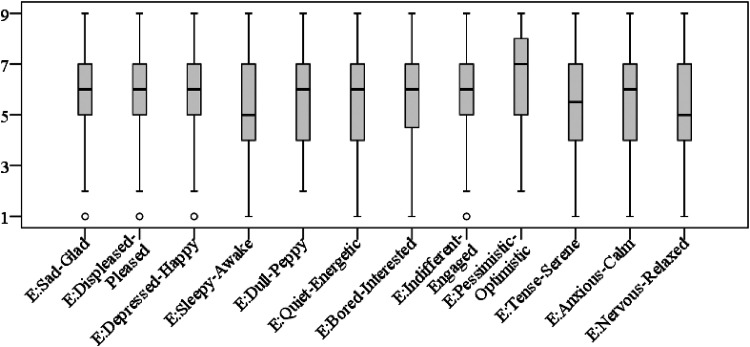
Participants’ moods before the experiment according to the Swedish Core Affect Scale.

First, we tested whether the participants’ emotional state differed significantly when experimental groups were compared. The Kruskal–Wallis test results indicated that the emotional state of participants in all three experimental groups did not differ significantly (depending on emotion pair the *p* values varied from .099 [Bored–Interested) to .812 (Sad–Glad)]. Their mood ratings are depicted in Figure 10, which shows that, in general, the participants reported mood with positive valence on all 12 bipolar adjective scales.

#### Facial Emotion Rating

**Figure 11.**
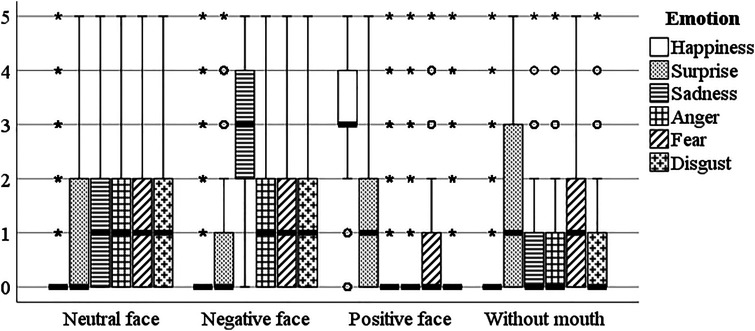
Average emotion ratings for different facial expressions.

In the facial emotion rating task, the highest scores refer to happy emotions for a positive facial expression (by which we mean stimuli with a convexity of the mouthline) and sad emotions for a negative facial expression (by which we mean stimuli with a concavity of the mouthline) (see Figure 11 and Table 2). For neutral facial expressions, the ratings of emotions typically are low, and the tendency is that negative emotions (sad, anger, fear, and disgust) have higher scores than positive emotions (happiness, surprise). These negative emotions have, on average, the same rates for stimuli with a negative facial expression, except for the sad emotion, which has a higher rating for negative facial expression. For positive facial expressions, emotions other than happiness are typically not recognized (sadness, anger, disgust) or rated low on average (surprise and fear). Although emotion ratings for stimuli without a mouth were low, relatively higher rates were found for surprise and fear.

#### The Effect of Instructions on Facial Emotion Rating

To see the impact of instruction, we compared the evaluations of each emotion between experimental groups, analyzing all stimuli together for each facial expression. Tables 3–5 summarize the mean rates in each group, and the significant differences (*p *< .05) according to Mann–Whitney tests are indicated. According to our results the instruction to observe sad faces increased the average rating scores for all emotions (the only exceptions were (a) happiness and surprise relating to positive facial expressions, (b) sad emotions relating to negative facial expressions, and (c) higher-rated emotions of stimuli without a mouth (fear and surprise). In contrast to what we expected, the instruction to observe happy faces did not increase the rating for positive emotions when a neutral face was presented but increased the scores for anger and disgust. In the case of negative facial expressions, the instruction to observe happy faces slightly reduced the average scores for all emotions (except disgust), and there was a similar trend for stimuli without a mouth. The instruction to observe happy faces reduced the scores for positive emotions (happiness and surprise) for positive facial expressions.

**Table 3.** Average emotion ratings compared between group given no instructions and group instructed to observe sad faces.

	Neutral face			Negative face			Positive face			Without a mouth		
	*M*		*U-*test *z* score	*M*		*U*-test *z* score	*M*		*U*-test *z* score	*M*		*U*-test *z* score
	i:n	i:s		i:n	i:s		i:n	i:s		i:n	i:s	
Happiness	0.3	0.6	−6.61***	0.2	0.5	−10.48***	3.3	2.9	−3.62***	0.4	0.6	−2.27*
Surprise	1.1	1.2	−1.88	0.6	0.8	−4.43***	1.4	1.3	−0.01	1.4	1.4	−0.04
Sadness	1.3	1.6	−3.40**	3.3	3.2	−0.34	0.2	0.7	−11.46***	0.7	0.9	−1.25
Anger	1.1	1.4	−4.10***	1.3	1.4	−3.08**	0.2	0.7	−12.29***	0.6	0.7	−1.58
Fear	1.3	1.4	−1.31	1.3	1.5	−3.11**	0.4	0.8	−9.61***	1.3	1.3	−0.03
Disgust	1.1	1.8	−8.10***	1.3	1.5	−3.43**	0.3	0.9	−13.67***	0.6	0.9	−2.05*

*Note*. i:n = Group given no instructions; i:s = Group instructed to observe sad faces.

*p < .05, **p < .01, ***p < .001.

**Table 4.** Average emotion ratings compared between group given no instructions and group instructed to observe happy faces.

	Neutral face			Negative face			Positive face			Without a mouth		
	*M*		*U*-test *z* score	*M*		*U*-test *z* score	*M*		*U*-test *z* score	*M*		*U*-test *z* score
	i:n	i:h		i:n	i:h		i:n	i:h		i:n	i:h	
Happiness	0.3	0.3	−0.26	0.2	0.1	−0.08	3.3	3.0	−2.93**	0.4	0.3	−1.31
Surprise	1.1	0.9	−1.79	0.6	0.5	−1.33	1.4	0.8	−6.80***	1.4	1.2	−1.43
Sadness	1.3	1.1	−2.26*	3.3	3.0	−2.04*	0.2	0.2	−0.64	0.7	0.5	−1.03
Anger	1.1	1.2	−1.14	1.3	1.1	−1.80	0.2	0.3	−2.15*	0.6	0.5	−0.44
Fear	1.3	0.9	−4.70***	1.3	1.0	−3.68***	0.4	0.3	−0.34	1.3	1.1	−1.09
Disgust	1.1	1.5	−4.62***	1.3	1.4	−2.15*	0.3	0.4	−4.00***	0.6	0.4	−0.81

*Note*. i:n = Group given no instructions; i:h = Group instructed to observe happy faces.

*p < .05, **p < .01, ***p < .001.

**Table 5.** Average emotion ratings compared between group instructed to observe happy faces and group instructed to observe sad faces.

	Neutral face			Negative face			Positive face			Without a mouth		
	*M*		*U*-test *z* score	*M*		*U*-test *z* score	*M*		*U*-test *z* score	*M*		*U*-test *z* score
	i:h	i:s		i:h	i:s		i:h	i:s		i:h	i:s	
Happiness	0.3	0.6	−5.32***	0.1	0.5	−7.63***	3.0	2.9	−0.48	0.3	0.6	−2.90**
Surprise	0.9	1.2	−3.02**	0.5	0.8	−4.75***	0.8	1.3	−6.07***	1.2	1.4	−1.39
Sadness	1.1	1.6	−4.57***	3.0	3.2	−1.38	0.2	0.7	−8.14***	0.5	0.7	−1.87
Anger	1.2	1.4	−2.30*	1.1	1.4	−4.05***	0.3	0.7	−7.53***	0.5	0.7	−1.70
Fear	0.9	1.4	−4.77***	1.0	1.5	−5.47***	0.3	0.8	−7.52***	1.1	1.3	−0.93
Disgust	1.5	1.8	−2.63**	1.4	1.5	−0.92	0.4	0.9	−7.08***	0.4	0.9	−2.34

*Note*. i:h = Group instructed to observe happy faces; i:s = Group instructed to observe sad faces.

*p < .05, **p < .01, ***p < .001.

#### The Effect of Facial Features on Facial Emotion Rating

When testing the impact of various facial features, we only analyzed emotions with average ratings 1 or greater than 1 in each facial expression group because 0 (according to the rating scale) relates to no perceived emotion and 1 relates to very weak perceived emotion. The reference faces’ average emotion ratings for each facial expression are provided in Table 6.

**Table 6.** Average emotion rating scores of reference face stimulus in each facial expression group.

Emotion	Facial expression			
	Neutral (reference face)	Negative (reference face)	Positive (reference face)	Without a mouth (mask, no mouth)
Happiness	0.3	0.3	3.1	0.4
Surprise	1.0	0.6	1.3	1.4
Sadness	1.3	3.1	0.3	0.7
Anger	1.0	1.2	0.4	0.6
Fear	1.1	1.3	0.5	1.3
Disgust	1.3	1.3	0.5	0.6

The Friedman test indicated significant differences for all emotions in the facial expression categories when stimuli with different facial features were compared. The three highest- and lowest-rated features are listed in Table 7 (for the ratings for all stimuli, see Appendix B). The significant differences mostly refer to comparisons of the stimuli with the highest and lowest ratings. However, Table 7 indicates the statistically significant differences (*p *< .05) from the reference face according to the Wilcoxon pairwise comparison test with Bonferroni correction.

**Table 7.** Average emotion rating scores for reference face and the three highest- and lowest-rated facial features in the facial expression categories (*p* values indicate significant differences from the reference face).

Facial expression	Emotion	Lowest ratings	Reference face	Highest ratings
Neutral	Surprise	0.6 EyesNarrow	1.0 Reference	2.6 EyesWide (*p *< .001)
		0.7 Body		1.2 MouthSmall
		0.8 Oval		1.1 MouthFar
	Sadness	0.8 EyesWide	1.3 Reference	1.6 MouthFar
		1.2 MouthClose		1.6 Round
		1.2 MouthThick		1.5 Body
	Anger	0.9 EyesWide	1.0 Reference	1.5 EyesNarrow
		1.0 MouthSmall		1.4 Body
		1.0 MouthFar		1.2 MouthClose
	Fear	0.9 EyesNarrow	1.1 Reference	2.2 EyesWide (*p = *.001)
		1.0 Body		1.4 MouthThin
		1.0 Oval		1.4 EyesClose
	Disgust	0.9 EyesWide	1.3 Reference	1.9 EyesNarrow
		1.0 EyesFar		1.6 Body
		1.1 MouthThick		1.4 MouthClose
Negative	Sadness	2.7 MouthMin	3.1 Reference	3.6 MouthFar
		2.9 EyesWide		3.5 MouthMax
		2.9 EyesNarrow		3.4 Round
	Anger	1.0 MouthFar	1.2 Reference	1.6 EyesNarrow
		1.1 EyesWide		1.5 MouthClose
		1.1 EyesFar		1.4 Oval
	Fear	1.0 Oval	1.3 Reference	2.1 EyesWide (*p = *.011)
		1.1 EyesNarrow		1.3 MouthFar
		1.1 Body		1.2 EyesClose
	Disgust	1.2 MouthFar	1.3 Reference	2.1 EyesNarrow (*p = *.038)
		1.2 EyesWide		1.6 MouthClose
		1.2 MouthThick		1.6 MouthLarge
Positive	Happiness	2.7 MouthMin	3.1 Reference	3.6 EyesNarrow
		2.8 MouthFar		3.5 Rabbit
		2.8 MouthThin		3.5 MouthMax
	Surprise	0.9 Body	1.3 Reference	2.3 EyesWide (*p = *.002)
		1.0 Round		1.4 MouthMax
		1.1 Oval		1.4 MouthThick
Without mouth	Surprise			1.4 Mask
				1.4 NoMouth
	Fear	1.1 Mask		1.4 NoMouth

Regarding variations of facial features, the most pronounced effects relate to the wide eyes stimulus, which significantly increases ratings for surprise if linked to neutral and positive facial expressions and fear for neutral and negative facial expressions. The narrow eyes stimulus shows highest ratings regarding anger and disgust for neutral and negative faces and happiness for positive facial expressions. However, it should be noted that unlike other eye variations, both features (wide eyes and narrow eyes) also correspond to facial expressions, not just facial geometry (that is, one can make one's eyes narrower or wider but cannot move them closer together or farther apart). For stimuli with mouth variations, the highest and lowest ratings for positive and negative facial expressions for happiness and sadness are associated with maximum and minimum curvature, although they did not show statistical significance with respect to the reference stimuli. These features are determined more by emotional expression than facial geometry.

#### Impact of Gender on Facial Emotion Rating

We also tested the impact of gender by comparing the emotion scores of male and female participants by facial expression. The data show that, on average, male participants in the group with no instruction rated emotions’ intensity higher than female participants (except sadness for negative facial expressions and surprise and fear for stimuli without a mouth) (Table 8).

**Table 8.** Average emotion ratings in each experimental group relative to different facial expressions.

Gender	Emotions	Neutral face			Negative face			Positive face			Without mouth		
		i:n	i:h	i:s	i:n	i:h	i:s	i:n	i:h	i:s	i:n	i:h	i:s
Female	Happiness	0.2	0.2	0.3	0.1	0.1	0.3	3.2	3.1	2.7	0.3	0.2	0.5
	Surprise	0.9	1.0	1.1	0.5	0.5	0.8	1.2	0.7	1.0	1.5	1.1	1.5
	Sadness	1.3	1.4	1.5	3.3	3.3	3.3	0.1	0.3	0.5	0.6	0.4	0.5
	Anger	0.8	1.2	1.3	1.0	1.2	1.1	0.1	0.3	0.6	0.5	0.5	0.5
	Fear	1.2	1.0	1.3	1.1	0.9	1.4	0.3	0.4	0.7	1.4	1.4	1.3
	Disgust	0.9	1.7	1.9	1.1	1.5	1.4	0.2	0.6	0.8	0.4	0.5	0.8
Male	Happiness	0.6	0.5	1.0	0.4	0.1	0.8	3.3	2.7	3.3	0.6	0.4	0.6
	Surprise	1.3	0.9	1.3	0.8	0.5	0.9	1.9	1.1	1.7	1.3	1.4	1.3
	Sadness	1.4	0.8	1.6	3.1	2.6	3.3	0.4	0.2	0.9	0.8	0.6	1.1
	Anger	1.5	1.0	1.4	1.8	1.0	1.8	0.4	0.3	0.9	0.7	0.6	0.9
	Fear	1.4	0.8	1.6	1.5	1.1	1.7	0.5	0.2	1.0	1.2	0.5	1.1
	Disgust	1.5	1.3	1.6	1.8	1.4	1.6	0.4	0.3	0.9	0.8	0.3	0.8

*Note*. i:n = Group given no instructions; i:h = Group instructed to observe happy faces; i:s = Group instructed to observe sad faces.

When comparing these tendencies between experimental groups, the GLM univariate analysis indicated significant (*p *< .05) interaction effects between gender and experimental group for each emotion and facial expression (Tables 9–12). Exceptions were found for fear for negative faces and surprise for positive faces, where both factors were significant but had no interaction effects, and the no mouth category, where there were no interaction effects.

**Table 9.** GLM results for different emotions by gender and instruction group (NEUTRAL facial expression).

	Rated emotions											
	Happiness		Surprise		Sadness		Anger		Fear		Disgust	
Variables	*F*	*η* ^2^	*F*	*η* ^2^	*F*	*η* ^2^	*F*	*η* ^2^	*F*	*η* ^2^	*F*	*η* ^2^
Gender	102.31***	.05	3.29	.00	1.57	.00	10.78**	.01	2.15	.00	0.05	.00
Group	15.97***	.02	4.08*	.00	9.64***	.01	3.32*	.00	16.65***	.02	21.21***	.02
Gender × Group	4.22*	.00	3.73*	.00	11.53***	.01	18.75***	.02	2.88	.00	23.75***	.02

*p < .05, **p < .01, ***p < .001.

**Table 10.** GLM results for different emotions by gender and instruction group (NEGATIVE facial expression).

	Rated emotions											
	Happiness		Surprise		Sadness		Anger		Fear		Disgust	
Variables	*F*	*η* ^2^	*F*	*η* ^2^	*F*	*η* ^2^	*F*	*η* ^2^	*F*	*η* ^2^	*F*	*η* ^2^
Gender	60.57***	.03	5.38*	.00	18.43***	.01	34.55***	.02	16.50***	.01	12.68***	.01
Group	40.92***	.04	10.88***	.01	7.05***	.01	8.38***	.01	14.36***	.01	.055	.00
Gender × Group	17.15***	.02	5.79**	.01	7.48***	.01	17.55***	.02	0.90	.00	16.13***	.02

*p < .05, **p < .01, ***p < .001.

**Table 11.** GLM results for different emotions by gender and instruction group (POSITIVE facial expression).

	Rated emotions											
	Happiness		Surprise		Sadness		Anger		Fear		Disgust	
Variables	*F*	*η* ^2^	*F*	*η* ^2^	*F*	*η* ^2^	*F*	*η* ^2^	*F*	*η* ^2^	*F*	*η* ^2^
Gender	3.26	.00	67.10***	.03	28.81***	.01	21.95***	.01	5.32*	.00	0.00	.00
Group	15.84***	.01	30.85***	.03	65.78***	.06	57.11***	.05	46.45***	.04	60.45***	.05
Gender × Group	12.56***	.01	2.95	.00	16.56***	.02	6.78**	.01	8.82***	.01	11.70***	.01

*p < .05, ** *p *< .01, ***p < .001.

**Table 12.** GLM results for different emotions by gender and instruction group (WITHOUT MOUTH stimuli).

	Rated emotions											
	Happiness		Surprise		Sadness		Anger		Fear		Disgust	
Variables	*F*	*η* ^2^	*F*	*η* ^2^	*F*	*η* ^2^	*F*	*η* ^2^	*F*	*η* ^2^	*F*	*η* ^2^
Gender	2.12	.01	0.01	.00	3.84	.01	2.13	.01	4.37	.02	0.14	.00
Group	1.01	.01	0.23	.00	1.06	.01	0.25	.00	1.09	.01	1.99	.02
Gender × Group	0.33	.00	0.39	.00	0.59	.01	.026	.00	0.93	.01	1.18	.01

Note: no significant effects were found.

Although the effect sizes from GLM analysis indicated small effects (as η² value), the comparison with the average rating scores (Table 8) showed interesting tendencies. For male participants, the tendency for all emotions was that the instruction to observe happy faces induced lower emotion ratings than the group given no instructions (the only exception was surprise relating to stimuli without a mouth). For female participants, such an instructional impact corresponded only for fear regarding neutral and negative facial expressions and happiness and surprise for positive facial expressions and expressions without a mouth (in this category, the sadness score also decreased with the instruction to observe happy faces). As a result, in contrast to the group given no specific instruction, most female ratings are higher than male ratings when analyzing the experimental group with the instruction to view happy faces.

For female participants, the instruction to observe sad faces typically induced higher ratings when compared to the group given no instructions except for positive emotions (happiness and surprise) for positive facial expressions and fear for stimuli without a mouth. This same tendency was also found among male participants. As a result, similar to the experimental group given no instructions, the male ratings in the experimental group with the instruction to observe sad faces are higher than the female participants’ ratings overall.

To sum up, the results indicate that the facial expression set by mouth curvature (positive or negative) is the determining factor in assigning the emotion of happiness or sadness. On average, the other emotions had low ratings for the tested stimuli (Figure 11). The variations in facial features may strengthen or weaken emotion intensity ratings; however, they do not change the general tendencies of the evaluations (Table 7).

When the impact of instructions is considered, the overall tendency is that the instruction to observe sad faces tends to increase emotion intensity ratings. The exceptions are emotions with the highest average rates: happiness and surprise for positive expressions, sadness for negative expressions, and fear and surprise for the “no mouth” category (Tables 3–5). The instruction to observe happy faces tends to reduce emotion intensity rates for negative facial expressions and stimuli without a mouth. However, regarding neutral and positive facial expressions, the impact is not typically unidirectional and varies depending on the emotion.

Further analysis of the impact of gender revealed the interaction effects with instruction type, which were not present just for stimuli without a mouth. In the experimental group with no instructions, male participants rated emotions’ intensity higher on average (Table 8). When instructed to observe happy faces, this tendency was reversed; overall, the instruction reduced the intensity of male participants’ ratings, but this was not typical of female participants. In turn, the instruction to observe sad faces mostly induced higher emotion ratings both for male and female participants, with the exception of surprise and happiness for positive facial expressions and a few emotions relating to stimuli with no mouth.

## General Discussion and Conclusions

Experiment 1 indicated that although there are longer and more frequent fixations on the eyes than on the mouth, and the first saccades are also directed to the eyes than to the mouth more frequently, the valence-containing instructions (to observe happy or sad faces) significantly increased the fixation time, number of fixations, and first saccade direction in favor of the mouth. At the same time, there was a decrease in fixation durations on the eyes once a task to observe particular emotions was introduced.

Our results indicate that initial saccadic directions and fixations are focused on the eyes by default. This occurs because of automatic capturing of attention (which might automatically induce fixation on the eyes). Eyes as attractors of attention are important for establishing interaction and attentional alignment between participants and occurs even in cases when less than the entire face is visible (Dalmaso et al., 2021). This also has an evolutionary explanation: establishing gaze contact is primary with respect to other facial information. Consistently with Dalmaso et al. (2017), our results indicate that early eye contact impacts further saccadic generation processes.

If separate facial features are considered, there are much subtler impacts (and no general trends can be determined). However, in general, valence-related instructions again increase fixations on the mouth area (although, in total, the area around the eyes is fixated on first and more frequently) (on the role of mouth and eyes, see also Blais et al., 2012; on valence, see Liu et al., 2020). In the case of individual features, a clear impact of the whole body condition arises: fixation duration decreases in the eye area and increases in the mouth area. Also, in the case of the face mask stimulus, fixation duration decreases in the eye area and increases in the mouth area, which most likely indicates the importance of the mouth for reading emotions on a partially covered face. Other impacts of individual factors are more heterogeneous and might be more difficult to interpret, for example, line drawings might decrease the overall configurational effects (Leder, 1996) impacting emotion reading; differences in configurational geometry (e.g., distance between eyes and mouth), the color of eyes are just a few factors that might impact emotion reading in partially covered faces.

Overall, experiment 1 indicates that observing faces with a particular valence-related instruction significantly increases the impact of mouth (duration, number, and sequence of fixations). Although the eyes are still fixated most frequently and with a longer fixation on the eyes, and first saccades are also directed toward the eyes, looking for emotions significantly increases fixation measurements relating to the mouth. (For differential valence-related effects with respect to eyes or mouth, see also Blais et al. (2012), Eisenbarth and Alpers (2011), and Liu et al. (2020))

We have to take into account that the current stimuli involved a very limited amount of visual information that could help in emotion recognition—the stimuli included only the eyes and the mouth, and did not include eyebrows, cheeks or any wrinkles that would aid in emotion recognition. Additionally, in real faces there are additional emotion cues complemented with discourse-related information (e.g., voice and gesturing) that impacts the resulting emotion reading. Therefore, the current findings specifically address the role of eyes and mouth in the emotion perception and should be interpreted with some caution.

Experiment 2 allowed us to evaluate the importance and impact of the mouth on emotion recognition. The scores for the reference face clearly indicate that the mouth's curvature (up or down) determines the detection of happy or sad emotions. The absence of the mouth or a neutral expression (mouth without curvature) did not allow particular emotions to be assigned to the face stimuli, and the average ratings were low. This fits with the results of Freud et al. (2020) and Grundmann et al. (2021).

We used variations of facial features to analyze in detail which facial components would have the strongest impact on emotion perception. Although our data confirmed that variations of facial features could increase or decrease the ratings for emotion intensity, these variations did not change the general tendencies. The significant differences generally refer to the comparisons of the geometric variation stimuli with the highest and lowest ratings when each experimental group and particular expressions are considered (Appendix B).

Significant differences from the reference face occurred for eyes that were wide open or tightly closed, which may be linked with the emotion expressed or with geometric characteristics of the face. Depending on the mouth's curvature, the respective stimuli were linked with surprise (wide eyes, positive and neutral expressions), fear (wide eyes, negative and neutral expressions), happiness (narrow eyes, positive expression), and anger and disgust (negative and neutral expression) (Table 7).

Regarding the stimuli with mouth variations, a mouth located far from the eyes increased ratings for sadness, and a mouth close to the eyes increased ratings for disgust and anger. Variations in the distance between the eyes (being closer or more distant from one another) had smaller effects on the intensity rating, and the differences from the reference stimuli rates were also smaller. This might indicate that horizontal alignment is more important for inducing emotions (Pachai et al., 2018). However, more experiments are needed for conclusive evidence.

Just as in the first experiment, we detected a significant effect from valence-containing instruction. The data show that the impact of instructions to observe happy or sad faces was not the same on the emotion intensity rating, as the latter instruction typically increased the average scores. However, the effect was different for the highest-rated emotions for negative expressions (sadness), positive expressions (surprise, happiness), and stimuli without a mouth (surprise, fear). The tendency in both cases was that the emotion intensity ratings were reduced or remained the same compared with the group given no instructions.

Otherwise, the instruction to observe happy faces did not have the same typically uniform impact as can be observed with the instruction to observe sad faces. This might be at least partially linked with interaction effects that we found between gender and instruction type. In contrast to what was expected, the male participants’ emotion ratings were generally higher than those of female participants. However, the instruction to observe happy faces reduced the average intensity rating for male participants, but this was not typical of female participants. Therefore, in this experimental group, female participants’ emotion ratings were often higher than those of male participants.

Our results also indicate that partially covered faces impair emotion reading, most likely by disturbing the holistic processing of facial information, where a covered mouth seems to be a critical missing component (see also Farah et al., 1998; Freud et al., 2020; Maurer et al., 2002; Rhodes, 1988). When the mouth is missing as a core source for emotion reading, observers seem to focus more on the eyes instead. This is reflected in the results of experiment 1. Furthermore, experiment 2 showed that it was difficult for participants to assign emotions when eyes correspond to reference configuration and the mouth is not visible.

To sum up both our studies, both the eyes and mouth are crucial in face perception for simple facial stimuli, but they have different functions and processing dynamics. Mouth perception seems to be a later-stage phenomenon, which can be explained by the evolutionary need to establish gaze contact first. The role (fixation frequency, fixation duration, and the relative frequency of the first fixation) of the mouth increases once there is a top-down instruction to observe a valence in the face. This means that the mouth's role increases in cases of emotion perception (relative to default face observation). Instructions impact the ratings of emotion intensity; however, the impacts are not uniform and differ depending on facial expression valence, instruction valence, rated emotion valence, and the gender of the rater.

Both experiments indicate that instruction-driven and valence-related attention impact the perception of facial information in both early (experiment 1, see the analysis of saccadic processes) and later (experiment 2) stages of processing. Our results support the idea that face perception is both a stage-wise and a holistic process, where primary facial information processing (establishing gaze contact) is followed by facial expression and emotion reading (increasing the perceptual load on the mouth area). Our results also at least partially support the argument that featural and configural processes in face perception run interdependently (see Tanaka & Sengco, 1997).

In our study, we are using line drawings representing structural relations between facial components, and experimentally modulating them. This has particular constraints and limitations. For instance, eyes in real life situations can convey more fine-grained information (e.g., color and pattern of gaze contact). Also, changes in the real-time processing during interaction also shape emotion recognition. Although there is eye-tracking evidence (Eisenbarth & Alpers, 2011) from real face pictures that both face and mouth contribute to emotion recognition, and that there are differences with respect to particular emotions, there are no studies where these differences are examined by accurately modulating facial features in line-drawn faces. Finally, of course, line drawings have their limits. We are aware that the line drawings might reduce configurational processing (Leder, 1996).

Future studies using a similar experimental design might be useful to check whether underlying geometry expressed in lines or abstract geometric objects—including in the absence of facial stimuli—emotionally constrain human reactions (e.g., Larson et al., 2012).

## Appendix B. Average Ratings for All Stimuli Arranged in Descending Order Within Categories

Rating scale: 0—no, 1—very weak, 2—weak, 3—average, 4—strong, and 5—very strong
